# Predictors of mortality in COPD exacerbation cases presenting to the respiratory intensive care unit

**DOI:** 10.1186/s12931-021-01657-4

**Published:** 2021-03-04

**Authors:** Yang Cao, Zhenzhen Xing, Huanyu Long, Yilin Huang, Ping Zeng, Jean-Paul Janssens, Yanfei Guo

**Affiliations:** 1 Department of Respiratory and Critical Care Medicine, Beijing Hospital, Beijing, China; 2 National Center of Gerontology, Beijing, China; 3 Institute of Geriatric Medicine, Chinese Academy of Medical Sciences, Beijing, China; 4 National Clinical Research Center for Respiratory Diseases, Beijing, China; 5 Department of Epidemiology, Institute of Geriatrics, Beijing Hospital, Beijing, China; 6 Division of Pulmonary Diseases, Department of Medicine, Geneva University Hospitals, Geneva, Switzerland

**Keywords:** Acute exacerbation of chronic obstructive pulmonary disease, In-hospital mortality, Predictors

## Abstract

**Background:**

Studies report high in-hospital mortality of acute exacerbation of chronic obstructive pulmonary disease (AECOPD) especially for those requiring admission to an intensive care unit. Recognizing factors associated with mortality in these patients could reduce health care costs and improve end-of-life care.

**Methods:**

This retrospective study included AECOPD patients admitted to the respiratory intensive care unit of a tertiary hospital in Beijing from Jan 1, 2011 to Dec 31, 2018. Patients demographic characteristics, blood test results and comorbidities were extracted from the electronic medical record system and compared between survivors and non-survivors.

**Results:**

We finally enrolled 384 AECOPD patients: 44 (11.5%) patients died in hospital and 340 (88.5%) were discharged. The most common comorbidity was respiratory failure (294 (76.6%)), followed by hypertension (214 (55.7%)), coronary heart disease (115 (29.9%)) and chronic heart failure (76 (19.8%)). Multiple logistic regression analysis revealed that independent risk factors associated with in-hospital mortality included lymphocytopenia, leukopenia, chronic heart failure and requirement for invasive mechanical ventilation.

**Conclusions:**

The in-hospital mortality of patients with acute COPD exacerbation requiring RICU admission is high. Lymphocytes < 0.8 × 10^9^/L, leukopenia, requirement for invasive mechanical ventilation, and chronic heart failure were identified as risk factors associated with increased mortality rates.

## Introduction

Chronic obstructive pulmonary disease (COPD) is a worldwide public health challenge because of high prevalence and related disability and mortality. World Health Organization projections predict that COPD-related mortality and disability will continue to increase worldwide until at least 2030 [1]. Acute exacerbation of COPD (AECOPD), defined as the worsening of respiratory symptoms and requirement of additional clinical treatment, tends to be a critical factor leading to poor outcome [2]. Exacerbation of COPD reduces lung function and quality of life, and is accompanied by an increased disease-related burden and a high hospital mortality [2–4]. Several studies have identified factors independently associated with in-hospital mortality due to COPD exacerbations, including cardiac dysfunction, duration of hospital stay, older age, comorbidities and nutritional status, and arterial oxygen and carbon dioxide partial pressure at entry. However, independent prognostic factors differ between studies [5–8]. In addition, few studies specifically targeted patients admitted in respiratory intensive care unit (RICU). The purpose of our study was to determine hospital mortality rate and factors affecting mortality for patients with AECOPD requiring RICU admission.

## Methods

### Study design and subjects

The electronic medical records of all patients aged ≥ 40 years admitted to the RICU with a diagnosis of AECOPD from Beijing Hospital during the period Jan 1, 2011 and Dec 31, 2018 were actively reviewed. AECOPD was defined as worsening of dyspnoea accompanied by increased cough and/or sputum volume or sputum purulence that results in additional therapy [2]. All diagnoses, including primary and five secondary diagnoses, were defined according to the International Classification of Diseases, 10th Revision (ICD10) coding system. Patients were excluded from the study if they were COPD patients admitted for diagnoses other than AECOPD. Patients whose duration of hospitalization was less than 24 h, and those re-admitted within 1 month were also excluded. Laboratory results obtained within 24 h of admission were used in this study. The criteria for the management of AECOPD in the RICU did not change during the study period.

### Data collection

Data were collected from the electronic medical record system, including demographic characteristics, routine blood tests, biochemical tests, arterial blood gases, pulmonary function tests and comorbidities. Demographic characteristic included age, gender, body mass index, smoking status, use of long-term home oxygen therapy, index of activities of daily living at admission, requirement for invasive mechanical ventilation and ventilator use duration. Blood tests included red cell count and white differential cell count, neutrophil-to-lymphocyte ratio, platelet-to-lymphocyte ratio, C reactive protein, N-terminal pro-brain natriuretic peptide, D-dimers, creatinine, uric acid. All blood samples were collected within 24 h after admission. Comorbidities recorded were: respiratory failure, hypertension, coronary heart disease (CHD) and chronic heart failure (CHF), atrial fibrillation, diabetes, and chronic kidney disease. Criteria for admission of AECOPD to the RICU did not change during the study period. The endpoint of the research was all-cause hospital mortality.

### Statistical analysis

All data were analyzed by using SPSS 25.0 (IBM, NY, USA). Continuous variables were expressed as mean and standard deviation (SD). Clinical data are expressed as mean ± SD for continuous variables then compared by t-tests. Data for categorical variables were presented as counts and proportions, and were compared by χ^2^ tests. The collinearity was tested between variables and the variance inflation factor (VIF) was less than 10 indicating no collinearity between variables. Univariable and multivariable logistic regression analysis were used to explore the risk factors associated with in-hospital mortality for AECOPD. We also considered different cutoff values such as p-value < 0.25, but gender and age were still not associated with in-hospital mortality as independent variables. Variables in univariate analyses with a p value < 0.1 were finally included in the multivariable logistic regression analysis to identify the independent risk factors for in-hospital mortality. A p-value < 0.05 was considered statistically significant.

## Results

Between January 2011 and December 2018, 452 patients were admitted to the RICU for AECOPD. After excluding 4 patients with less than 24 h of hospitalization, 26 patients who were readmitted within one month and 38 patients with unavailable key data in the electronic medical record, we included 384 patients in the final analysis. Forty-four patients (11.5%) died during their hospital stay, and 340 patients (88.5%) were discharged.

### Clinical features and comorbidities in patients

The present research included data of 384 AECOPD patients admitted to the RICU. Patients were divided into survivors and non-survivors based on in-hospital mortality. Demographic characteristics and baseline data of patients in the survival and non-survival groups are presented in Table 1. The average age of all patients was 78.2 ± 8.2 years. There were more males (72.9%) than females (27.1%). Patients who died in the hospital had a lower ADL index at admission (28.6 ± 28.9) than survivors (43.1 ± 30.0, p = 0.003). The ventilation duration time was longer in non-survivors (438.3 ± 505.3 h) than in survivors (269.7 ± 317.0 h, p = 0.042). Requirement for IMV was significantly higher in the non-survivor group (65.9% vs. 7.1%, p < 0.001). The most common complication was respiratory failure (76.6%), followed by hypertension, CHD and CHF. Patients with CHF as a comorbidity were more frequent in non-survivors than survivors (50.0% vs. 15.9%, p < 0.001).

**Table 1:** Clinical features and comorbidities in all patients included, in survivors and in non-survivors

Variables	Total (N = 384)	Survivors (n = 340)	Non-survivors (n = 44)	P value
Age, years	78.2 (8.2)	78.0 (8.2)	79.7 (7.6)	0.185
Gender				
Male	280 (72.9%)	244 (71.8%)	36 (81.8%)	0.158
Female	104 (27.1%)	96 (28.2%)	8 (18.2%)	
BMI, kg/m^2^	23.2 (5.3)	23.3 (5.2)	22.7 (5.8)	0.579
Smoking status				
Never smoker	85 (22.3%)	77 (22.8%)	8 (18.2%)	0.073
Former smoker	242 (63.4%)	208 (61.5%)	34 (77.3%)	
Current smoker	55 (14.4%)	53 (15.7%)	2 (4.5%)	
Smoking exposure, pack-years	35.6 (34.5)	35.3 (35.0)	38.3 (30.8)	0.593
Long-term home oxygen therapy	76 (19.8%)	68 (20.0%)	8 (18.2%)	0.776
ADL index at admission	41.3 (30.2)	43.1 (30.0)	28.6 (28.9)	0.003
Requirement for IMV	53 (13.8%)	24 (7.1%)	29 (65.9%)	< 0.001
Ventilator use time, hours	294.2 (354.5)	269.7 (317.0)	438.3 (505.3)	0.042
Comorbidities				
Respiratory failure	294 (76.6%)	258 (75.9%)	36 (81.8%)	0.382
Hypertension	214 (55.7%)	194 (57.1%)	20 (45.5%)	0.145
CHD	115 (29.9%)	102 (30.0%)	13 (29.5%)	0.951
CHF	76 (19.8%)	54 (15.9%)	22 (50.0%)	< 0.001
Atrial fibrillation	64 (16.7%)	55 (16.2%)	9 (14.1%)	0.474
Diabetes	97 (25.3%)	85 (25.0%)	12 (27.3%)	0.744
CKD	55 (14.3%)	47 (13.8%)	8 (18.2%)	0.437

Date are presented as mean ± standard deviation (SD) for continuous variables and percentages for categorical variables

*BMI* body mass index, *IMV* invasive mechanical ventilation, *CHF* chronic heart failure, *CHD* coronary heart disease, *CKD* chronic kidney diseases

### Laboratory examination of patients on admission

Table 2 shows laboratory results on admission. In non-survivors, mean leukocyte counts were higher (10.2 ± 6.3 × 10^9^/L, vs. 8.2 ± 3.3 × 10^9^/L, p = 0.043), lymphocyte counts were lower (0.84 ± 0.89 × 10^9^/L vs. 1.09 ± 0.60 × 10^9^/L, p = 0.020), and less patients had lymphocytopenia (lymphocytes < 0.8 × 10^9^/L: 33.4% vs 65.9%) than in survivors. NLR and PLR calculated from absolute numbers of lymphocytes, neutrophils and platelets (Table 2), were higher in non-survivors. CRP, NT-proBNP, and D-dimer were also significantly higher in non-survivors than in survivors. Conversely, albumin and PaCO_2_ were lower in non-survivors. No significant differences in lung function were noted.

**Table 2:** Laboratory results of patients within 24 h after admission

Variables	Total (N = 384)	Survivors (n = 340)	Non-survivors (n = 44)	P value
White blood cell count, × 10^9^/L	8.4 (3.8)	8.2 (3.3)	10.2 (6.3)	0.043
< 4	21 (5.5%)	16 (4.7%)	5 (11.4%)	0.002
4–10	269 (70.1%)	248 (72.9%)	21 (47.7%)	
> 10	94 (24.5%)	76 (22.4%)	18 (40.9%)	
Neutrophil count, × 10^9^/L	7.3 (7.8)	7.1 (8.0)	8.8 (6.4)	0.187
Lymphocyte count, × 10^9^/L	1.05 (0.65)	1.09 (0.60)	0.84 (0.89)	0.020
< 0.8	141 (37.2%)	112 (33.4%)	29 (65.9%)	< 0.001
≥ 0.8	243 (62.8%)	228 (66.6%)	15 (34.1%)	
Platelet count, × 10^9^/L	187.2 (81.1)	188.3 (79.0)	178.7 (96.5)	0.460
< 100	40 (10.4%)	31 (9.1%)	9 (20.5%)	0.021
≥ 100	348 (89.6%)	309 (90.9%)	35 (79.5%)	
NLR, %	10.8 (17.3)	9.6 (16.7)	19.9 (19.5)	< 0.001
PLR, %	253.1 (355.0)	240.8 (357.0)	362.4 (332.3)	0.033
Eosinophil count, × 10^9^/L	0.12 (0.19)	0.11 (0.14)	0.15 (0.40)	0.213
CRP, mg /L	5.1 (7.0)	4.7 (6.7)	8.4 (8.6)	0.002
Albumin, g/L	35.2 (5.5)	35.4 (5.4)	33.5 (5.9)	0.030
NT-proBNP, pg/ml	1123.3 (2586.0)	948.0 (2280.2)	2474.3 (4066.3)	0.039
D-dimers, ug/L	726.6 (1075.8)	676.5 (1018.4)	1127.0 (1392.9)	0.009
Creatinine, umol /L	83.4 (63.1)	81.0 (60.4)	102.2 (79.6)	0.099
Uric acid, umol /L	260.5 (131.9)	254.9 (126.0)	304.1 (166.2)	0.067
pH	7.37 (0.07)	7.37 (0.07)	7.39 (0.06)	0.186
PaO_2_, mmHg	78.0 (25.9)	77.9 (23.2)	78.3 (41.4)	0.960
PaCO_2_, mmHg	52.8 (15.8)	53.5 (16.1)	47.7 (12.5)	0.022
PaO_2_/FiO_2_, mmHg	275.7 (85.5)	277.59 (83.9)	260.7 (96.7)	0.225
Post-bronchodilator FEV_1_^*^, L	0.94 (0.46)	0.95 (0.46)	0.82 (0.10)	0.705
Post-bronchodilator FVC^*^, L	1.95 (0.69)	1.95 (0.70)	1.77 (0.42)	0.714
Post-bronchodilator FEV_1_/FVC^*^, %	47.16 (12.39)	47.13 (12.39)	48.61 (17.19)	0.869

Date is presented as mean ± standard deviation for continuous variable and percentages for categorical variables

*NLR* neutrophil/lymphocyte ratio, *PLR* platet/lymphocyte ratio, *CRP* C reactive protein, *NT-proBNP* N-terminal pro-brain natriuretic peptide, *FEV*_*1*_ forced expiratory volume in 1 s, *FVC* forced vital capacity, *PaO*_*2*_ arterial partial pressure of oxygen, *PaCO*_*2*_ arterial partial pressure of carbon dioxide

*Data missing for Post-bronchodilator FEV_1_, FVC and FEV_1_/FVC (n = 93)

### Independent risk factors for in-hospital mortality

Requirement for IMV, the admission index of ADL, CHF, WBC, lymphocyte, platelet, NLR, CRP, albumin, NT-proBNP, D-dimer and PaCO_2_ were all significant variables in univariable analysis (Table 3), while age, gender, smoking status and PLR were not included in the logistic regression analysis. This analysis showed that requirement for IMV (OR = 30.31, 95% CI: 8.29–110.74, p < 0.001), CHF (OR = 7.63., 95% CI: 2,27–25.64, p = 0.001), leukopenia (OR = 5.77, 95% CI: 1.05–31.74, p < 0.044) and lymphocytopenia (OR = 3.60, 95% CI: 1.10–11.76, p = 0.034) were independent risk factors associated with in-hospital mortality.

**Table 3:** Logistic regression analysis of the in-hospital mortality of AECOPD requiring RICU admission

Variable	Univariable OR (95%CI)	P value	Multivariable OR (95%CI)	P value
Age, years	1.03 (0.99–1.07)	0.185		
Gender (M/F)	1.77 (0.79–3.95)	0.163		
Requirement for IMV	25.46 (12.04–53.83)	< 0.001	30.31 ( 8.29–110.74)	< 0.001
The admission index of ADL	0.98 (0.97–1.00)	0.004	0.99 (0.97 -1.02)	0.578
Smoking status (ever vs. never)	1.33 (0.59–2.98)	0.491		
CHF	5.30 (2.74–10.23)	0.001	7.63 (2.27–25.64)	0.001
White blood cell count, × 10^9^/L				
< 4	3.95 (1.31–11.94)	0.015	5.77 (1.05–31.74)	0.044
4–10	1.00 (ref)		1.00 (ref)	
> 10	2.81 (1.42–5.54)	0.003	3.05 (0.90–10.31)	0.073
Lymphocyte count, × 10^9^/L				
< 0.8	3.85 (1.98–7.47)	< 0.001	3.60 (1.10–11.76)	0.034
≥ 0.8	1.00 (ref)		1.00 (ref)	
Platelet count, × 10^9^/L				
< 100	2.56 (1.23–5.82)	0.025	1.64 (0.30–8.91)	0.652
≥ 100	1.00 (ref)		1.00 (ref)	
NLR, %	1.02 (1.01–1.04)	0.008	1.01 (0.99–1.03)	0.292
PLR, %	1.00 (1.00–1.00)	0.115		
CRP, mg /L				
< 10	1.00 (ref)		1.00 (ref)	
≥ 10	2.55 (1.21–5.45)	0.016	5.57 (0.99–31.30)	0.051
Albumin, g/L				
< 35	1.93 (1.00–3.70)	0.049	1.81 (0.51–6.40)	0.356
≥ 35	1.00 (ref)		1.00 (ref)	
NT-pro BNP, pg/L	1.00 (1.00–1.00)	0.014	1.00 (1.00–1.00)	0.163
D-dimer, ug/L				
< 500	1.00 (ref)		1.00 (ref)	
500–1000	2.08 (0.98–4.42)	0.057	1.07 (0.26–4.33)	0.931
> 1000	2.97 (1.33–6.63)	0.008	1.97 (0.44–8.65)	0.369
PaCO_2_, mmHg	0.97 (0.95–1.00)	0.026	0.99 (0.94–1.05)	0.508

*IMV* invasive mechanical ventilation, *CHF* chronic heart failure, *NLR* neutrophil/lymphocyte ratio, *PLR* platet/lymphocyte ratio, *NT-proBNP* N-terminal pro-brain natriuretic peptide, *CRP* C reactive protein

## Discussion

The present study identified several risk factors for death in adults who were hospitalized with AECOPD in a respiratory ICU (RICU). In particular, lymphocytes < 0.8 × 10^9^/L, leukopenia, requirement for IMV, and having CHF were associated with higher odds of in-hospital death.

Knowledge about prognosis of disease and factors that predict poor outcome is important to help physicians to advise patients on the expected natural course of an illness. Several risk factors that predict death from AECOPD have been identified in prior studies. For instance, C-reactive protein (CRP), neutrophil–lymphocyte ratio (NLR), platelet-lymphocyte ratio (PLR), value of D-dimers and N-terminal-pro hormone B-type natriuretic peptide (NT-pro BNP) have been reported as being associated with in-hospital mortality in AECOPD patients [9–14]. To the best of our knowledge, predicting in-hospital mortality of ICU patients with AECOPD based on lymphocytopenia has been reported in only a few studies. Acanfora et al. showed that relative a lymphocyte count ≤ 20% was associated with higher risk of death in elderly patients with moderate-to-severe COPD [15]. Xiong et al. and Yao et al. noted that patients with COPD who died had a lower lymphocyte count than patients who survived, but lymphocyte count was not an independent risk factor for in-hospital mortality of AECOPD patients [11, 16]. We observed that lymphocytopenia occurred in more than 65% of non-survivors and was an independent risk factor for in-hospital mortality (OR 3.60 (1.10–11.76)).

Mechanisms for lymphocytopenia predicting high risk of in-hospital death in patients with AECOPD remains unclear. Several facts should be considered. First, peripheral blood lymphocytes may be decreased in the elderly [17, 18] and older age was also a significant risk factor for COPD mortality as reported in previous studies [19, 20]. Second, a lower lymphocyte count as a biomarker of inflammation could increase the risk of infection increasing risk of death from AECOPD. Lymphocytopenia was found in the critically ill patients with SARS-CoV infection because targeted invasion by SARS-CoV viral particles damages the cytoplasmic component of the lymphocytes and causes their destruction [21]. Additionally, lymphocytopenia resulting from apoptosis of lymphocytes is also common in severe MERS infection [22]. In the present study, lymphocyte count was shown to be a useful, widely available, and inexpensive predictor that may help identify AECOPD patients admitted to the RICU at high risk of in-hospital mortality.

Requirement for IMV was a significant predictor of in-hospital mortality of AECOPD [20, 23]. In a study by Brown et al., 38.7% of patients required IMV; multivariate analysis showed that requirement for IMV was strongly associated with in-hospital death [20]. Lindenauer PK et al. showed that in-hospital mortality was higher in COPD patients who required IMV than in patients with non-invasive ventilation (NIV) [24]. The results of the present study were consistent with previous studies. This finding is not surprising: typically, patients who require IMV rather than NIV are in a severe disease stage.

CHF is a common comorbidity of COPD [25]. In the present study, having CHF as a comorbidity was an important risk factor for predicting in-hospital mortality of AECOPD patients. Testa et al. found that patients with COPD and CHF had an increased risk of mortality compared with patients with either COPD or CHF alone [26]. The results of the present study were consistent with their study. There are some potential pathophysiological mechanisms that could explain the interaction between COPD and cardiovascular disease. With the progression of COPD, the increased pulmonary vascular resistance leads to pulmonary hypertension and right ventricular dysfunction. In addition, both hypoxia and acidosis can reduce diastolic and systolic myocardial dysfunction [27, 28].

Our study also has several limitations. Firstly, the results may not be generalizable to other ICU patients because of a single-center design. Secondly, management of respiratory insufficiency did not follow a prospective protocol and the individual preferences of the treating physician may have affected outcome. Thirdly, we do not have precise information on nutritional status or quality of life prior to admission. Fourthly, we had no detailed information about COPD severity (CAT, mMRC) because the included subjects are severe patients. And only 93 participants reported spirometry parameters owing to severe patients failure to cooperatively complete lung function test. Finally, the study could not provide post-hospital mortality data, which would be necessary for further validation of the prognostic factors in our findings.

Hospitalization for acute COPD exacerbation is becoming more frequent, and it places an enormous burden on patients and health care systems. In conclusion, the current study has identified a number of variables associated with in-hospital mortality for AECOPD patients in RICU.

## Data Availability

The datasets during and/or analysed during the current study available from the corresponding author on reasonable request.
